# Protein Reporter Bioassay Systems for the Phenotypic Screening of Candidate Drugs: A Mouse Platform for Anti-Aging Drug Screening

**DOI:** 10.3390/s120201648

**Published:** 2012-02-07

**Authors:** Takuya Chiba, Tomoshi Tsuchiya, Ryoichi Mori, Isao Shimokawa

**Affiliations:** 1 Department of Investigative Pathology, Graduate School of Biomedical Sciences, Nagasaki University, 1-12-4 Sakamoto, Nagasaki 852-8523, Japan; E-Mails: ryoichi@nagasaki-u.ac.jp (R.M.); shimo@nagasaki-u.ac.jp (I.S.); 2 Division of Surgical Oncology, Graduate School of Biomedical Sciences, Nagasaki University, 1-7-1 Sakamoto, Nagasaki 852-8501, Japan; E-Mail: tomoshi@nagasaki-u.ac.jp

**Keywords:** drug development, high throughput screening, reporter mice, age-related disorders

## Abstract

Recent drug discovery efforts have utilized high throughput screening (HTS) of large chemical libraries to identify compounds that modify the activity of discrete molecular targets. The molecular target approach to drug screening is widely used in the pharmaceutical and biotechnology industries, because of the amount of knowledge now available regarding protein structure that has been obtained by computer simulation. The molecular target approach requires that the structure of target molecules, and an understanding of their physiological functions, is known. This approach to drug discovery may, however, limit the identification of novel drugs. As an alternative, the phenotypic- or pathway-screening approach to drug discovery is gaining popularity, particularly in the academic sector. This approach not only provides the opportunity to identify promising drug candidates, but also enables novel information regarding biological pathways to be unveiled. Reporter assays are a powerful tool for the phenotypic screening of compound libraries. Of the various reporter genes that can be used in such assays, those encoding secreted proteins enable the screening of hit molecules in both living cells and animals. Cell- and animal-based screens enable simultaneous evaluation of drug metabolism or toxicity with biological activity. Therefore, drug candidates identified in these screens may have increased biological efficacy and a lower risk of side effects in humans. In this article, we review the reporter bioassay systems available for phenotypic drug discovery.

## 1. Introduction

Reporter assays have been widely used in biological research [1–3]. Such assays are a powerful means of investigating the signaling pathways involved in various biological functions induced by the activation of specific transcription factors. The reporter construct usually consists of a promoter (enhancer) that drives the transcription of a gene that is easily detected by its activity or expression in the assay system. The promoter region binds transcription factors that are activated in response to the stimulation of an upstream, receptor-mediated or receptor-independent signaling cascade. Binding of the transcription factor to the promoter results in the induction of reporter gene (and subsequent reporter protein) expression in response to signaling pathway activation by external stimuli, including drug candidates. It is important that the exogenous protein encoded by the reporter gene has unique enzymatic activity or another property that distinguishes it from endogenous proteins [4].

The activity of the reporter protein is typically assayed by photometry, colorimetry or fluorometry. Recently, detection systems based on bioluminescence, chemiluminescence and fluorescence have become widely used because of their signal detection sensitivity and ease of use. Improved detection equipment, such as charge-coupled device (CCD) cameras, has also enabled the development of convenient reporter assay systems for drug screening. There are two major types of reporters, *i.e.*, intracellular and extracellular reporters. Intracellular reporter gene products are expressed and retained inside the cells. On the other hand, extracellular reporter genes encode proteins that are secreted into the medium of cultured cells or the blood stream in animals. Some intra- and extracellular reporter gene assays may detect the reporter protein’s activity without killing the cells or animals in which the assay is performed. This allows time-course experimentation by sampling of the medium of cultured cells, or the blood plasma of research subjects. As such, report bioassays are potentially suitable for high throughput screening (HTS) of small molecule drug candidates. Bioassays are used to determine the concentration or biological activity of molecules such as hormones, growth factors, and enzymes. Moreover, they can be used for measuring the effects of candidate drugs on an organism, cultured cells, or recombinant receptors by comparison to gold standard molecules with known target activity. The present review provides an overview of the basic properties, efficacies and limitations of protein reporter assays, with an emphasis on their application as bioassays for drug screening.

## 2. Intracellular Reporters

### 2.1. Chloramphenicol Acetyltransferase

Chloramphenicol *O*-acetyltransferase (CAT) has, until recently, been widely used as a reporter protein [5]. CAT is a bacterial enzyme that is not expressed in eukaryotes, and it therefore provides a highly specific detection system. CAT catalyzes the reaction between acetyl-CoA and chloramphenicol to form CoA. The CAT assay has been widely used but is difficult to adapt to HTS platforms because its activity is not easy to detect directly in cells.

### 2.2. β-Galactosidase

β-Galactosidase (β-Gal) is derived from the bacterial lacZ gene and is widely used as an intracellular reporter gene. There are a number of detection options depending on which substrate is used. However, β-Gal is affected by endogenous enzymatic activity in most mammalian cells [4]. Therefore, it is important to distinguish between the endogenous and exogenous (reporter) enzymes in the assay system. This usually requires the pH of the assay mixture to be adjusted, although this may not completely eliminate endogenous enzyme activity. Therefore, the utility of β-Gal for HTS assays is limited.

### 2.3. Luciferase

Genes encoding luciferase enzymes have been cloned from several species, including firefly and sea pansy [1]. Firefly luciferase catalyzes the oxidation of firefly luciferin, which produces a short-lived flash of light that decays within a few seconds. However, the sensitivity of the luciferase assay is significantly higher than the CAT assay [6]. The high sensitivity of luciferase-based bioluminescence assays is better suited to HTS platforms than CAT or β-Gal assays. Moreover, various luciferase substrates are available that have a long half-life and provide linear results over eight orders of magnitude [4]. The enzyme has also been used in a dual-luciferase reporter assay system, which allows both firefly and sea pansy luciferase reactions to be monitored independently in the same cell [7]. This enables the activation of two different signaling pathways to be measured simultaneously in response to the same stimulus.

### 2.4. Green Fluorescent Protein

Rather than being a reporter enzyme, green fluorescent protein (GFP) (and its engineered derivatives) is particularly suited to bioimaging assays in living cells or live animals. GFP is produced by the jellyfish, *Aequorea victoria*. Upon excitation by UV light, GFP emits green light with an emission maximum at 509 nm [4]. The intensity of light emissions has been improved by protein engineering in the fluorophore region of the protein. GFP-based reporter assays provide a distinct advantage because they do not require cell permeabilization or the addition of exogenous substrates. Therefore, these proteins are suitable for drug screening in HTS assays. Recent improvements in GFP detection technologies allow changes in the localization of GFP-tagged proteins, in response to small molecules, to be detected at the subcellular level in living cells cultured in 384-well plates. Therefore, GFP is suitable as both a reporter of transcriptional activation, and of functional protein translocation in the cell, in response to various stimuli.

## 3. Extra-Cellular Reporters

### 3.1. Secreted Alkaline Phosphatase (SEAP)

The secreted alkaline phosphatase (SEAP) reporter system has been used to investigate the activity of known or putative promoter/enhancer elements [8]. As a reporter, SEAP has several important advantages over other reporters, such as high sensitivity and specificity both *in vitro* and *in vivo* [9–12]. SEAP is a C-terminal truncated mutant of the membrane-anchored, placental alkaline phosphatase [13]. Removal of the anchoring domain results in secretion of the enzyme into the culture medium or blood stream, thus enabling repeated sampling from living cells or animals. Expression of the gene can be detected using chemiluminometry [14]. The secreted form of alkaline phosphatase is extremely heat stable, and its activity can be detected following the inactivation of endogenous alkaline phosphatases by heating. Therefore, it is easy to distinguish between exogenous and endogenous SEAP activity. SEAP assays can also be coupled to a luciferase reaction by incubation with the substrate, firefly d-luciferin-*O*-phosphate. The product of the first reaction, luciferin, then becomes a substrate for luciferase. This assay protocol improves the sensitivity of the reporter assay system, thus enabling its broader application [15].

### 3.2. Secreted luciferase

A recently-identified secreted form of luciferase also serves as a useful reporter for HTS assays. Luciferase from the marine copepod, *Metridia longa,* has unique secretary features that make it suitable for extracellular reporter assays. Markova *et al.* reported the substrate specificity of *Metridia* luciferase (MetLuc) and demonstrated its suitability for use as a reporter for monitoring gene expression [16]. In common with SEAP, secreted MetLuc is well suited to monitoring gene expression in time-course reporter assays. Both SEAP and MetLuc are currently available in a commercial dual reporter system that has been successfully applied in drug screening research [17–20].

## 4. Phenotypic Screening

Reporter assay systems are suitable for the multi-parameter phenotype screening of drug candidates [21]. In contrast to the single molecular target approach, cell-based reporter assays reflect the complexity of the living organism. Therefore, they are becoming widely used to screen the effects of small molecules on signaling pathways [22]. However, drug discovery by HTS in cell-based assays presents its own difficulties. For instance, isolation of a hit compound often does not suggest a single, particular molecular target, preventing further optimization of hit compounds by medicinal chemistry [23]. This has led to cell-based screens being termed ‘black-box’ screens [21]. However, in most cases, target pathways may be predicted and investigated further. Nonetheless, drug companies prefer the well-characterized molecular target approach rather than phenotypic screening to accelerate the early phase of drug development. In fact, cell-based screens tend to identify more false positives than molecular target screens, presumably because of the existence of many potential targets in the biological pathway of interest [24]. Nonetheless, hit compounds from molecular target screens can fail to generate the expected effect on cells or have undesirable side effects that are potentially harmful to humans. In the context of the ongoing paradigm shift towards pathway-driven drug discovery, particularly in the academic field, it becomes increasingly important to accomplish promising candidate isolation earlier in the drug discovery pipeline and with higher throughput. Currently, many large pharmaceutical companies have established open innovation research programs through collaboration with non-profit research organizations to overcome these problems. Academic research teams typically utilize cell-based reporter assay systems for primary drug screening. Furthermore, research is also being conducted using living animals to monitor the more complex dynamics of the hit compounds identified by screening.

## 5. *In Vivo* Reporter Assays Using Mice

Animal-based reporter assays using reporter mice are a potentially powerful tool for the identification of drug candidates. To our knowledge, the ERE–Luc (estrogen responsive element–luciferase gene) mouse is the first example of a reporter animal to be used for drug development [25], although several studies with simple reporter mice have been documented previously [26–28]. The ERE–Luc model is a unique reporter animal for drug discovery that was developed to identify ligands that bind estrogen receptors (ERs). Because ERs are ubiquitously expressed in mammals, this mouse model was required to be generally applicable to the activity of ERs [29].

Validation of the animal model is important to establish whether the reporter activity correlates with receptor activation through the targeted pathways. Nonetheless, the ERE–Luc mouse demonstrated the existence of unliganded activation of ERs [30]. Thus, animal-based reporter assays may provide new insights into the physiology of target pathways. Indeed, the use of the reporter mice revealed differential mechanisms of ER activation in reproductive and non-reproductive tissues [31]. These results indicate that reporter mice are useful for biological research. Precise toxicology studies may also be performed by crossing another mouse model, such as the humanized liver metabolism model, with reporter mice [32]. Such studies might require that the high costs of compound provision are met, and that animal welfare conditions are appropriate. Table 1 summarizes the properties of reporter proteins.

## 6. Screening of Anti-Aging Drugs That Mimic the Beneficial Effects of Calorie Restriction

Calorie restriction (CR) extends the lifespan of many organisms [33]. The identification of CR mimetic compounds may therefore enable therapeutic retardation of the onset of various age-related disorders, including metabolic syndrome, cancers, neurodegenerative diseases, and other rare disorders [34]. The development of CR mimetics would therefore fulfill unmet medical needs. Although SIRT1-activating compounds (STACs) may offer a promising approach to the development of CR mimetics, a novel approach to finding potential CR mimetics is required [9]. For this purpose, we developed a cell- and mouse-based screening system using an extracellular reporter assay [10].

Using microarray analysis, we have identified putative regulatory elements in pro-longevity genes (dwarfism and CR-response elements: DFCR-RE) [9,35]. Using one of the DFCR-REs, we have developed a cell- and animal-based reporter screening system, named CRISP: the CR-Imitating anti-aging agent Screening Platform. Because the DFCR-REs were isolated from long-lived mice, the unidentified transcriptional regulators of this sequence may be beneficial for longevity and delay the onset of age-related diseases. We found that reporter construct transgenic mice (CRISP mice) showed increased reporter activity upon CR ([9] and our unpublished data). Hence, compounds that activate this reporter may be CR mimetic pro-longevity drug candidates. As revealed in the ERE-Luc mice, CRISP mice may also reveal novel longevity signaling pathways and target molecules. The activity of candidate compounds can be easily studied on cells by their addition to culture medium, or in mice by their addition to food and water. In addition to isolating the activators of pro-longevity elements, our CRISP method may also identify inhibitors or negative regulators of these elements. In this respect, CRISP is advantageous compared to single molecular target screening. Therefore, a two-step screen in which a primary HTS in CRISP cells is used to identify hit compounds, followed by a secondary screen to verify the activity, metabolism and toxicology of the hits in CRISP mice, is a potentially powerful tool for identifying promising novel drug candidates.

## 7. Conclusions

Current reporter assay technologies enabled us to perform high throughput screening of chemical libraries. The isolation of candidate molecules is dependent on the choice of reporter protein and the construction of efficient cell- or animal-based assays. The identification of CR mimetics is attractive for the development of anti-aging therapeutics. While CR mimetics may not be a ‘cure-all’ for age-associated disorders, they may suppress and/or delay the progression of various kinds of refractory diseases. Importantly, pharmaceutical companies do not have a high incentive to develop drugs for rare diseases. Therefore, academic laboratories are perfectly placed to develop drugs for rare disorders by using phenotypic cell- or animal-based reporter assay systems. Although candidate CR mimetics identified in such systems must still be validated to show that their beneficial effects are similar to those provided by CR, CR mimetics offer great potential to solve the unmet medical needs of rare diseases and common age-related disorders. We have focused on bioassay (protein reporter assay) screening systems in this review, but biosensor technologies (analytical devices consisting of biological components) are also important for drug discovery [36]. Therefore, the development of biosensors to screen CR mimetics will also be an important approach to future drug discovery efforts.

## Figures and Tables

**Table 1.** Properties of reporter proteins.

	**Specificity**	**Application to HTS**	**Time-course Assay^1^**	***In vivo* Assay^2^**	**Specific Tissue^3^**	**Sensitivity to CCD camera^4^**
CAT	B	C	C	C	C	C
β-Gal	B	C	C	C	C	C
Luciferase	A	A	B	B	A	A
GFP	A	A	B	B	A	B
SEAP	A	A	A	A	B	A
MetLuc	A	A	A	A	B	A

A: suitable, B: applicable, C: not applicable.

1 Time-course assay is suitable for extra-cellular reporters compared to intra-cellular reporters.

2 An *in vivo* imaging system is needed to detect luciferase and GFP.

3 A tissue specific transgenic mouse is needed to detect specific tissue expression of extra-cellular reporters.

4 Chemiluminescence is more sensitive than fluorescence. CAT: Chloramphenicol acetyltransferase; β-Gal: β-Galactosidase; GFP: green fluorescent protein; SEAP: secreted alkaline phosphatase; MetLuc: Metridia longa luciferase; HTS: high throughput screening; CCD: charge-coupled device.
